# Dr. Thomas H. Musgrove

**Published:** 1895-07

**Authors:** 


### DR. THOMAS H. MUSGROVE.

DIED at Philadelphia, April 1, 1895, of pneumonia, Thomas H. Musgrove, D.D.S., in the sixty-third year of his age.

Dr. Musgrove was born in Newark, New Castle County, Delaware, September 7, 1832. He received his early education at the academy in his native town. When about eighteen or nineteen years of age he began the study of dentistry with Drs. Haines and McKorran, of Newark, Delaware. After completing his pupilage he located in Tuscaloosa, Alabama, where he remained but a year in consequence of ill health. Returning north, he located in Elkton, Maryland, where he pursued his professional work until the breaking out of the Civil War in 1861. Dr. Musgrove cast his lot with the Southern Confederacy, and in company with two companions succeeded in reaching Richmond, where he joined the Army of Northern Virginia. He remained in that service, experiencing many hardships, until the autumn of 1864, when, hearing of the serious illness of his aged father, he resolved to reach home at any risk. Obtaining a furlough to visit the far South, he turned his face northward instead, and after many weary days and nights of travel, much of the way on foot, he succeeded in reaching Washington and Baltimore. He was arrested in Baltimore as a rebel spy, was thrown into prison, and kept in close confinement until the spring of 1865, when, through the influence of friends, his case was presented in all its details to President Lincoln. His pardon was signed, and he was liberated but a few weeks before Mr. Lincoln's assassination. Dr. Musgrove was a brave soldier, and during his service in the Confederate ranks endured hardships which would break the spirit of men less courageous than he.

At the close of the war he again located in Elkton, Maryland, and continued in practice there until 1872, when he removed to Philadelphia and associated himself with the late Dr. Daniel Neall, with whom he remained for a period of four years. He then established himself in practice, and enjoyed the confidence and respect of all who sought his services until the day of his death. Dr. Musgrove was a modest, retiring man, a true friend, a devoted son to his widowed mother, a loving brother, and an honest man.

